# Association between body composition and the risk of mortality in the obese population in the United States

**DOI:** 10.3389/fendo.2023.1257902

**Published:** 2023-11-24

**Authors:** Heeso Lee, Hye Soo Chung, Yoon Jung Kim, Min Kyu Choi, Yong Kyun Roh, Jae Myung Yu, Chang-Myung Oh, Joon Kim, Shinje Moon

**Affiliations:** ^1^ Department of Family Medicine, Hallym University College of Medicine, Chuncheon, Republic of Korea; ^2^ Division of Endocrinology and Metabolism, Department of Internal Medicine, Hallym University College of Medicine, Chuncheon, Republic of Korea; ^3^ Department of Biomedical Science and Engineering, Gwangju Institute of Science and Technology, Gwangju, Republic of Korea

**Keywords:** sarcopenia, obesity, muscle, obesity paradox, mortality

## Abstract

**Background:**

Recent studies have presented the concept of the obesity paradox, suggesting that individuals with obesity have a lower risk of death than those without obesity. This paradox may arise because body mass index (BMI) alone is insufficient to understand body composition accurately. This study investigated the relationship between fat and muscle mass and the risk of mortality in individuals with overweight/obesity.

**Methods:**

We used data from the National Health and Nutrition Examination Survey (NHANES) from 1999 to 2006 and 2011 to 2018, which were linked to mortality information obtained from the National Death Index. Multiple Cox regression analyses were performed to estimate mortality risk. Subgroup analysis was conducted using propensity score-matched (PSM) data for age, sex, and race/ethnicity.

**Results:**

This study included 16,555 participants who were overweight/obese (BMI≥25 kg/m^2^). An increase in appendicular skeletal muscle mass index was associated with a lower mortality risk (hazard ratio [HR]: 0.856; 95% confidence interval [CI]: 0.802–0.915). This finding was consistent with the subgroup analysis of the PSM data. Contrastingly, a high fat mass index was associated with an increased risk of mortality. Sarcopenic overweight/obesity was significantly associated with high mortality compared to obesity without sarcopenia (HR: 1.612, 95%CI: 1.328–1.957). This elevated risk was significant in both age- and sex-based subgroups. This finding was consistent with the subgroup analysis using PSM data.

**Conclusion:**

In contrast to the obesity paradox, a simple increase in BMI does not protect against mortality. Instead, low body fat and high muscle mass reduce mortality risk.

## Introduction

In 2016, the World Health Organization reported that 13% of the global population was obese and that the obesity rate has increased threefold over the past 30 years (1). Obesity is associated with various diseases, including cardiovascular disease (CVD), diabetes, cerebral infarction, and cancer, and is responsible for approximately 4.8% of deaths worldwide (2–6). Although it is important to diagnose obesity accurately, recent studies have indicated an obesity paradox, which states that obese individuals may have a lower mortality risk than those with normal weight (a finding in two representative studies). A review of 40 cohort studies found that individuals with body mass indices (BMIs) <20 kg/m^2^ had a higher risk of total and cardiovascular mortality, whereas individuals who were overweight (BMI: 25–29.9 kg/m^2^) had a lower risk of mortality than those with a normal BMI (7). Furthermore, individuals who were overweight (BMI: 25–30 kg/m^2^) or obese (BMI: 30–35 kg/m^2^) and had hypertension and coronary artery disease had a lower risk of all-cause mortality than those with a normal weight (BMI: 20–25 kg/m^2^) (8). This paradox may be attributed to the use of BMI, which does not accurately measure body composition, to define obesity. To obtain precise measurements of body composition, including body fat and muscle mass, imaging tests such as computed tomography (CT), magnetic resonance imaging, dual-energy X-ray absorptiometry, and positron emission tomography/CT are required. However, these tests are expensive and not readily accessible. Therefore, BMI is commonly used to assess the severity of obesity. Nevertheless, BMI has limitations as it cannot accurately differentiate between fat, mineral, and muscle masses and has restrictions in measuring body fat (9–16). Consequently, BMI alone may not be sufficient for predicting the risk of CVD, diabetes, cerebral infarction, cancer, and mortality (17–19).

Although obesity is commonly believed to protect against sarcopenia by preserving muscle mass, it can impair muscle function and lead to functional limitations (20, 21). Moreover, sarcopenia can occur in individuals with obesity, resulting in sarcopenic obesity, in which individuals may have a high BMI but poor lean body mass, leading to increased disability, immobility, and metabolic dysfunction (22, 23). Due to its negative impact on quality of life, physical function, and mortality, the prevalence of sarcopenic obesity is concerning (24, 25). Therefore, clinicians and researchers need to recognize and address sarcopenic obesity as a distinct clinical entity to improve health outcomes and enhance the quality of life.

This study aimed to use data from the National Health and Nutrition Examination Survey (NHANES) to determine the prevalence of sarcopenic obesity and investigate the relationship between fat and muscle mass, particularly in relation to sarcopenic obesity and the risk of mortality.

## Method and materials

### Study population

The NHANES is a research program designed to assess the health and nutritional status of adults and children in the United States (26). This longitudinal study used baseline data from four NHANES cycles from 1999 to 2006 and 2011 to 2018, linked to mortality data from the National Death Index (27).

### Measurements

The average of three blood pressure (BP) measurements, taken after at least 5 minutes in a seated position, was used as data. Fasting blood glucose and cholesterol levels were measured by enzymatic methods. The NHANES Laboratory Procedure Manual provides additional information on sample collection and testing methods (28). Whole-body DEXA was performed using a Hologic QDR 4500 A fan-beam X-ray bone densitometer (Hologic Inc., Marlborough, MA, USA). DEXA was also used to assess total and regional body composition.

### Definition of obesity/overweight, underlying diseases, and sarcopenia

Obesity/overweight was defined as a BMI ≥25 kg/m^2^. Participants were considered to have hypertension if they had systolic BP >140 mmHg, mean diastolic BP >90 mmHg, or were receiving treatment for hypertension. Diabetes mellitus was defined as a fasting blood glucose level >126 mg/dL, random blood glucose level >200 mg/dL, HbA1c level >6.5%, or treatment for diabetes. Patients with a fasting total cholesterol level of 240 mg/dL or receiving treatment for dyslipidemia were considered to have dyslipidemia. Cancer history was assessed using structured questionnaires as follows: “*Have you ever been told by a doctor or other health professional that you have cancer or a malignancy?”* Patients with one or more of the following conditions were considered to have a history of CVD: angina pectoris, myocardial infarction, coronary heart disease, congestive heart failure, and cerebrovascular disease. Appendicular skeletal mass was defined as the sum of the total lean mass, excluding the bone mineral content of all extremities. The appendicular skeletal mass index (ASMI) was defined as the value obtained by dividing the appendicular skeletal mass by the square of the height (m) (29). We defined low muscle mass as ASMI <7 kg/m^2^ in men and <5.5 kg/m^2^ in women, according to the European Working Group on Sarcopenia in Older People 2 (EWGSOP2) (30). Sarcopenic obesity/overweight was defined as a BMI ≥25 kg/m^2^ with a low muscle mass. We also defined high fat mass as a fat mass index (FMI) ≥ 8.3 kg/m^2^ in men and ≥ 11.8 kg/m^2^ in women based on the study of Gonzalez et al. (31).

### Study outcomes

We collected publicly available mortality data from the National Center for Health Statistics based on probabilistic matching of the NHANES and National Death Index death certificate records through December 31, 2019. The data included all-cause mortality and follow-up duration through the month (27).

### Ethics statement

All the NHANES protocols conducted in the United States were authorized by the Research Ethics Review Board of the National Center for Health Statistics, U.S. Centers for Disease Control and Prevention (Protocol Number: 1999–2004, Protocol #98–12; 2005–2010, Protocol #2005–06; 2011–2016, Protocol #2011–17) and conducted in accordance with the principles of the Declaration of Helsinki. All participants provided informed consent prior to the study.

### Statistical analysis

Continuous and categorical variables of demographic characteristics, underlying diseases, anthropometric indices, and blood test results are presented as means, standard deviations, and frequencies (%), respectively. Independent t-tests and Pearson’s chi-square tests were used to compare results. Correlations between BMI, ASMI, and FMI were examined using Pearson’s correlation coefficient. Multiple Cox regression analyses were performed to assess the hazard ratios (HR) of all-cause mortality after adjusting for age, sex, race, smoking status, alcohol consumption, hypertension, diabetes, hyperlipidemia, and previous CVD events. The time from the first anthropometric and clinical measurements to death or the last follow-up (December 31, 2019) was included in the calculation. In addition, we performed a subgroup analysis of the propensity score matching (PSM) data (1:4 matching), considering the heterogeneity of the demographic characteristics and underlying diseases of sarcopenia. The graphical association between HR for each obesity parameter and mortality was assessed using four-knot-restricted cubic spline plots. Statistical analyses were performed using the IBM Statistical Package for the Social Sciences Statistics version 24.0 (IBM Corporation, Armonk, NY, USA) and R version 4.2.2 (R Foundation for Statistical Computing, Vienna, Austria; www.r-project.org). Statistical significance was set at p < 0.05.

## Results

### Baseline characteristics

**Figure 1:**
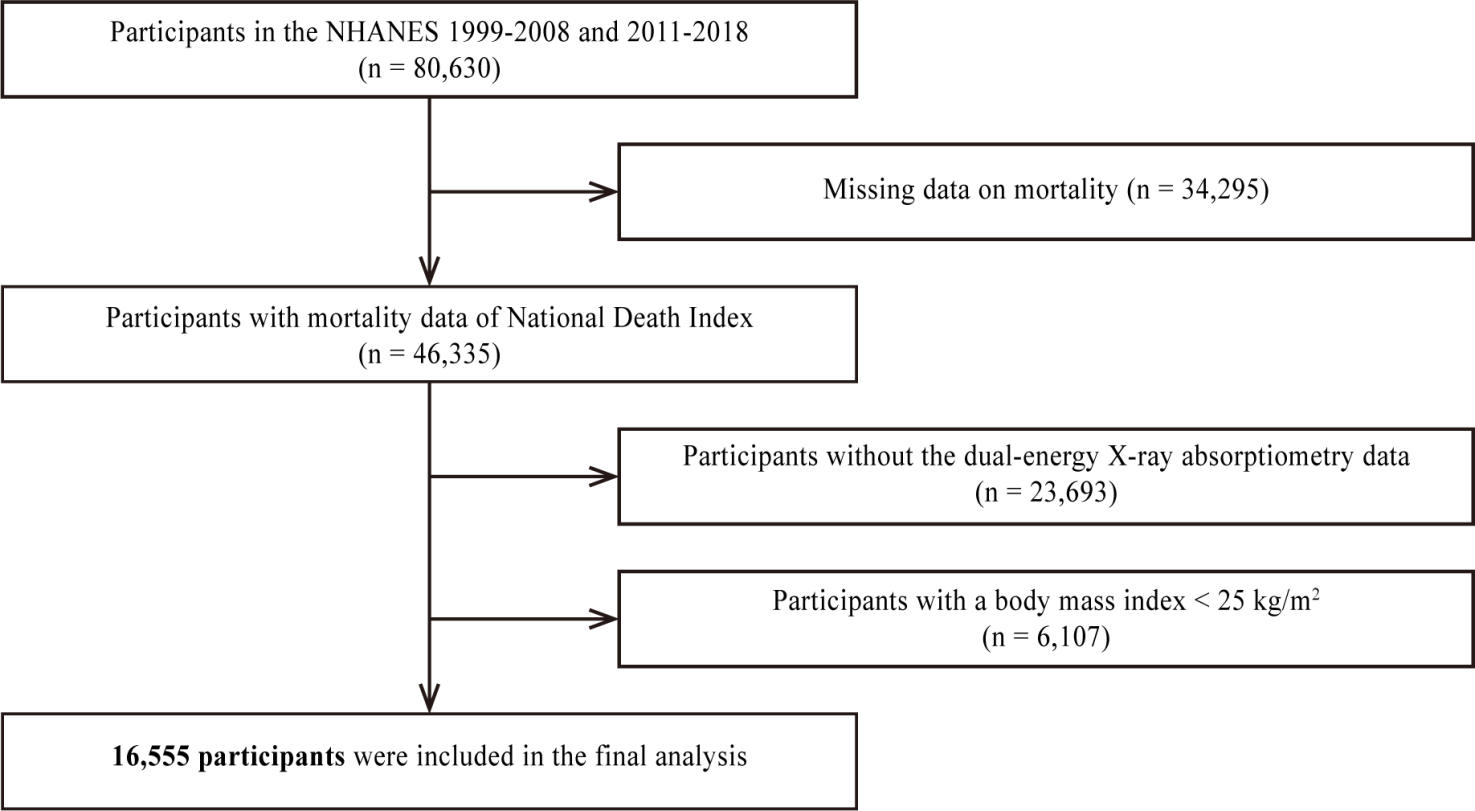
Flowchart for final selection. NHANES, National Health and Nutrition Examination Survey.

In this study, 80,630 participants were initially identified from the 1999–2006 and 2011–2018 NHANES datasets (**Figure 1**). After excluding 34,295 participants with missing mortality data, 23,693 with insufficient data, and 6,107 with BMIs <25 kg/m^2^, the final analysis included 16,555 participants. **Table 1** shows the baseline characteristics of the participants with obesity or overweight according to their sarcopenia status. The sarcopenia group (n=291) was older (61.7 ± 16.51 years), had a lower proportion of men (43.0%), and exhibited significant differences in race/ethnicity distribution compared to the group without sarcopenia (n=16,264). There were no significant differences in the triglyceride levels between the groups. The prevalence of smoking, cancer history, and systolic BP was higher, whereas the proportion of drinkers, BMI, and diastolic BP was lower in the sarcopenia group. Patients in the sarcopenia group had higher fasting glucose, HbA1c, total cholesterol, and high-density lipoprotein (HDL) cholesterol levels. In addition, the sarcopenia group had a higher prevalence of previous CVD, diabetes mellitus, hypertension, and dyslipidemia. The proportion of all-cause deaths was higher in the sarcopenia group. Finally, ASMI was lower in the sarcopenia group, whereas FMI was not significantly different between the two groups. BMI was positively correlated with ASMI (*Pearson correlation coefficient*: 0.524, p<0.001) and FMI (*Pearson correlation coefficient*: 0.831, p<0.001).

**Table 1:** Characteristics of whole and propensity score-matched participants with obesity/overweight according to sarcopenia.

	Whole dataset			PSM dataset		
	(N=16555)			(N=1385)		
	Without sarcopenia	Sarcopenia	p	Without sarcopenia	Sarcopenia	p
	(N=16264)	(N=291)		(N=1108)	(N=277)	
Age, years	42.97 ± 15.86	61.7 ± 16.51	< 0.001	62.2 ± 15.62	62.35 ± 15.70	0.883
Male sex, n (%)	8440 (51.9%)	125 (43.0%)	0.003	434 (39.2%)	121 (43.7%)	0.193
Race/Ethnicity, n (%)			< 0.001			< 0.778
Hispanic	5008 (30.8%)	111 (38.1%)		426 (38.4%)	106 (38.3%)	
Non-Hispanic White	6378 (39.2%)	155 (53.3%)		591 (53.3%)	151 (54.5%)	
Non-Hispanic Black	3565 (21.9%)	9 (3.1%)		42 (3.8%)	7 (2.5%)	
Other Race	1313 (8.1%)	16 (5.5%)		49 (4.4%)	13 (4.7%)	
Smokers, n (%)	6739 (43.5%)	160 (55.9%)	0.001	594 (53.6%)	154 (55.6%)	0.599
Drinkers, n (%)	10101 (68.0%)	166 (59.7%)	0.013	659 (59.5%)	166 (59.9%)	0.945
History of cancer, n (%)	839 (5.5%)	43 (15.0%)	< 0.001	150 (13.5%)	43 (15.6%)	0.436
BMI, kg/m^2^	31.03 ± 5.08	26.5 ± 1.2	< 0.001	30.23 ± 4.30	26.46 ± 1.11	< 0.001
Systolic BP, mmHg	123.27 ± 17.72	133.17 ± 23.60	< 0.001	132.95 ± 21.26	133.70 ± 23.54	0.626
Diastolic BP, mmHg	72.17 ± 12.56	70.01 ± 12.18	0.006	70.79 ± 13.47	70.13 ± 12.26	0.487
Fasting glucose, mg/dL	107.91 ± 37.09	117.71 ± 50.37	0.001	114.87 ± 41.84	118.26 ± 51.25	0.410
HBA1c, %	5.66 ± 1.07	5.89 ± 1.21	< 0.001	5.86 ± 1.13	5.91 ± 1.22	0.595
Total cholesterol, mg/dL	198.29 ± 42.62	209.5 ± 43.88	< 0.001	205.98 ± 40.59	209.86 ± 43.91	0.173
Triglycerides, mg/dL	161.56 ± 159.77	153.35 ± 88.04	0.512	167.03 ± 123.17	154.85 ± 89.44	0.249
HDL-C, mg/dL	48.93 ± 13.67	55.26 ± 18.36	< 0.001	50.61 ± 14.19	54.79 ± 16.95	< 0.001
Previous CVD, n (%)	1076 (7.1%)	61 (21.6%)	< 0.001	176 (16.0%)	59 (21.7%)	< 0.032
Diabetes Mellitus, n (%)	2172 (13.7%)	65 (22.9%)	< 0.001	236 (21.6%)	63 (23.2%)	0.622
Hypertension, n (%)	6222 (40.1%)	184 (65.7%)	< 0.001	664 (63.8%)	177 (66.5%)	0.444
Dyslipidemia, n (%)	5993 (45.9%)	162 (63.3%)	< 0.001	563 (59.0%)	157 (63.3%)	0.241
All-cause death, n (%)	1794 (11.0%)	141 (48.5%)	< 0.001	461 (41.6%)	136 (49.1%)	0.029
ASMI, kg/m^2^	8.38 ± 1.47	5.88 ± 0.75	< 0.001	7.50 ± 1.15	5.89 ± 0.75	< 0.001
FMI, kg/m^2^	11.27 ± 3.86	10.90 ± 1.80	0.103	11.76 ± 3.42	10.85 ± 1.72	< 0.001

BMI, body mass index; HBA1c, hemoglobin A1c; HDL-C, high-density lipoprotein cholesterol; CVD, cardiovascular disease; ASMI, appendicular skeletal mass index; FMI, fat mass index.

We also performed 1:4 PSM to minimize the impact of confounding variables in the data analysis. The factors accounted for in the PSM included diabetes mellitus, hypertension, dyslipidemia, previous CVD events, and ASMI or FMI, and they were matched for age, sex, ethnicity/race, smoking status, and alcohol consumption. **Table 1** shows the baseline characteristics of propensity score-matched (PSM) participants with obesity or overweight status according to their sarcopenia status. There were no differences between the groups in age, sex, race, or medical history, except for CVD. The prevalence of CVD and HDL cholesterol was higher in the sarcopenia group, whereas BMI, ASMI, and FMI were lower in the sarcopenia group.

### Association between body composition indices and mortality in participants with obesity/overweight

**Figure 2:**
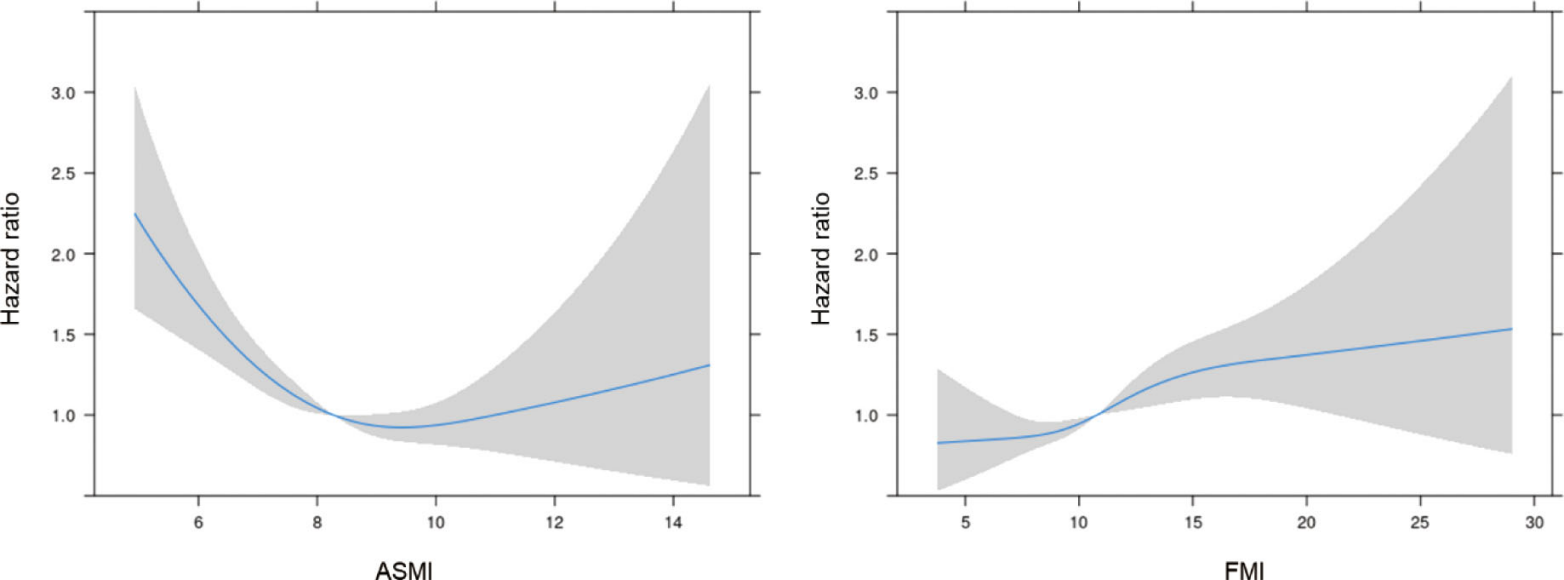
The hazard ratio for mortality according to ASMI and FMI. ASMI, appendicular skeletal mass index; FMI, fat mass index. Adjusted for age, sex, ethnicity/race, smoking status, alcohol consumption, history of cancer at baseline, diabetes mellitus, hypertension, dyslipidemia, previous CVD events, and ASMI or FMI.

According to the results of the multiple Cox regression analyses, an increase in ASMI was significantly associated with a reduced risk of mortality (HR: 0.856, 95% confidence interval [CI]: 0.802–0.915, p=0.001; **Table 2**). This negative association was observed in both age subgroups, i.e., those aged ≤ 65 years (HR: 0.836, 95% CI: 0.760–0.919, p<0.001) and > 65 years (HR: 0.875, 95% CI: 0.798–0.960, p=0.005; **Table 2**). When stratified by sex, the negative association remained significant in both men (HR: 0.826; 95% CI: 0.758–0.900; p<0.001) and women (HR: 0.897; 95% CI: 0.806–0.998; p=0.046; **Table 2**). When stratified by cause of death, ASMI was negatively associated with death from cancer (HR: 0.829; 95% CI: 0.726–0.947; p=0.006) and non-cancer causes (HR: 0.867; 95% CI: 0.803–0.935; p<0.001), but not with death from CVD/cerebrovascular accident (HR: 0.974; 95% CI: 0.867–0.973; p=0.659). We further analyzed the PSM dataset. This subgroup analysis confirmed a negative association between ASMI and all-cause mortality (HR: 0.899; 95% CI: 0.830–0.973; p=0.008; **Table 2**). In contrast, a higher FMI was significantly correlated with an increased risk of mortality. (HR: 1.045, 95% CI: 1.021–1.069, p<0.001; **Table 2**). This association was consistent in participants aged ≤ 65 years (HR: 1.064; 95% CI: 1.029–1.099; p<0.001) and > 65 years (HR: 1.033; 95% CI: 1.001–1.066; p=0.046; **Table 2**). Among men, a higher FMI significantly increased the risk of mortality (HR: 1.079; 95% CI: 1.043–1.116; p<0.001). However, this association was not significant in women (HR: 1.017, 95% CI: 0.984–1.051, p=0.325; **Table 2**). In contrast to ASMI, when stratified by cause of death, FMI was positively associated with cancer (HR: 1.057; 95% CI: 1.009–1.107; p=0.019) and non-cancer mortality (HR: 1.041; 95% CI: 1.014–1.069; p=0.003), but not CVD/cerebrovascular accident (HR: 1.027; 95% CI: 0.986–1.069; p=0.204). In the PSM dataset, contrary to the total dataset, FMI and mortality were negatively correlated (HR: 0.955; 95% CI: 0.925–0.985; p=0.004). In the restricted cubic spline regression plot, the ASMI showed a negative association with the HR for mortality. Conversely, the HR for mortality continuously increased with FMI (**Figure 2**).

**Table 2:** The risk of mortality according to the body composition indices in participants with obesity/overweight.

	ASMI		FMI	
	HR (95% CI)	P-value	HR (95% CI)	P-value
Total	0.856 (0.802–0.915)	<0.001	1.045 (1.021–1.069)	<0.001
Subgroup by age				
Aged ≤ 65 years	0.836 (0.760–0.919)	<0.001	1.064 (1.029–1.099)	<0.001
Aged > 65 years	0.875 (0.798–0.960)	0.005	1.033 (1.001–1.066)	0.046
Subgroup by sex				
Men	0.826 (0.758–0.900)	<0.001	1.079 (1.043–1.116)	<0.001
Women	0.897 (0.806–0.998)	0.046	1.017 (0.984–1.051)	0.325
Subgroup by cause of death				
Cancer	0.829 (0.726–0.947)	0.006	1.057 (1.009–1.107)	0.019
Non-cancer	0.867 (0.803–0.935)	<0.001	1.041 (1.014–1.069)	0.003
CVD/CVA	0.974 (0.867–1.094)	0.659	1.027 (0.986–1.069)	0.204
PSM data*	0.899 (0.830–0.973)	0.008	0.955 (0.925–0.985)	0.004

ASMI, appendicular skeletal mass index; FMI, fat mass index; CVD, cardiovascular disease; CVA, cerebrovascular accident.

Adjusted for age, sex, ethnicity/race, smoking status, alcohol consumption, history of cancer at baseline, diabetes mellitus, hypertension, dyslipidemia, previous CVD events, and ASMI or FMI.

*Adjusted for diabetes mellitus, hypertension, dyslipidemia, previous CVD events, and ASMI or FMI in propensity score-matched data for age, sex, ethnicity/race, smoking status, and alcohol consumption.

### The risk of mortality in sarcopenic obesity/overweight

**Figure 3:**
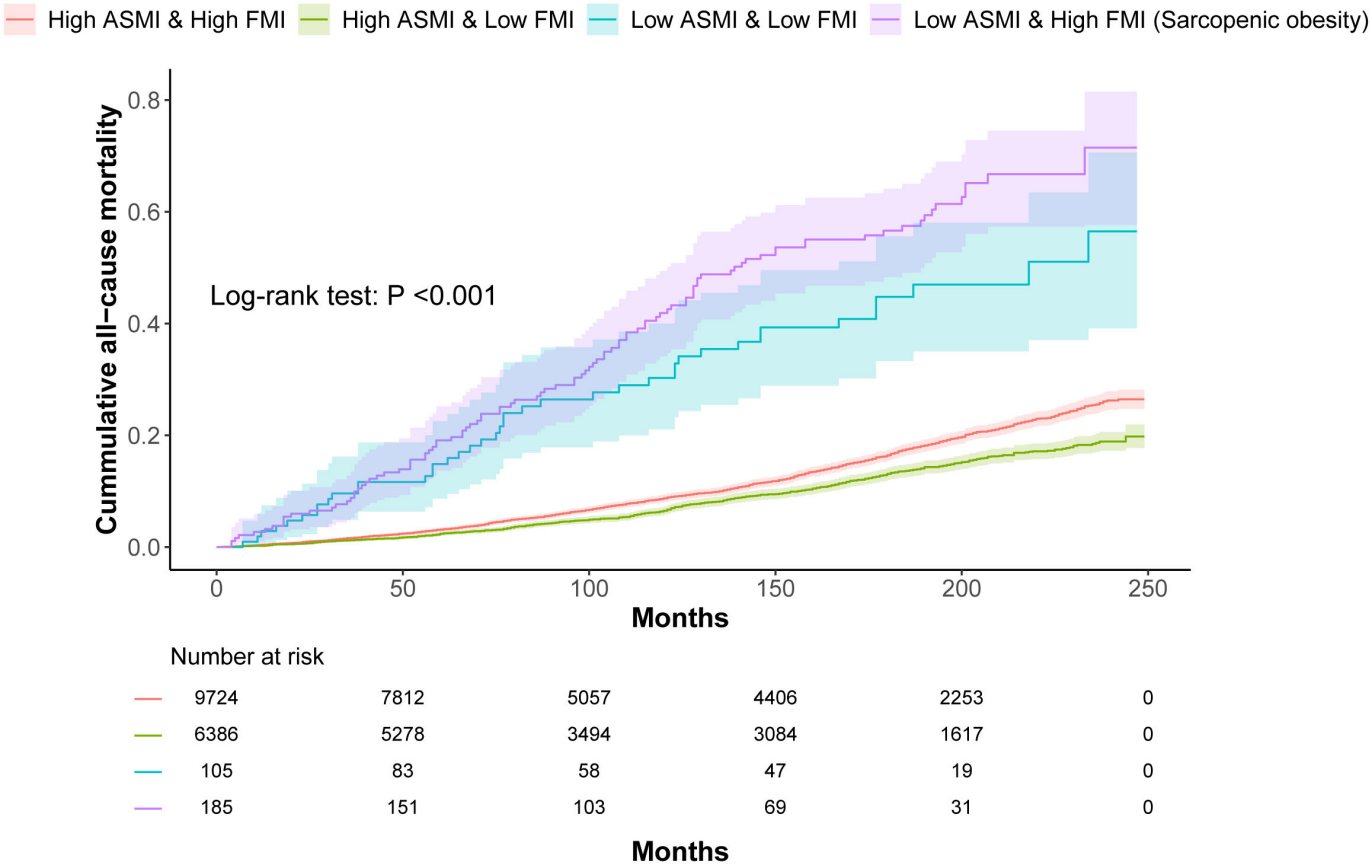
Kaplan–Meier plot of cumulative mortality by ASMI and FMI in participants with obesity.

Kaplan–Meier survival curves showed a relationship between ASMI, FMI, and mortality, with the highest mortality rates in the low ASMI and high FMI groups (log-rank test, p<0.001) (**Figure 3**).

**Table 3** presents the HR for mortality among individuals with sarcopenic obesity/overweight compared with those with obesity/overweight but sufficient ASMI, as determined by multiple Cox regression analyses. Sarcopenic obesity/overweight status was significantly associated with a higher risk of death. When analyzed by age subgroups, it was significantly associated with a higher risk of death in those ≤ 65 years of age (HR: 2.707; 95% CI: 1.813–4.043; p<0.001), but no significant association was found in those > 65 years of age (HR: 1.174; 95% CI: 0.982–1.404; p=0.079; **Table 3**). In the sex subgroups, the elevated risk was significant for both men and women (**Table 3**). In the subgroup analyses by cause of death, sarcopenic obesity/overweight was associated with an increased risk of cancer (HR: 1.541; 95% CI: 1.028–2.309; p=0.036) and non-cancer mortality (HR: 1.629; 95% CI: 1.306–2.031; p<0.001) but not CVD/cerebrovascular accident (HR: 1.367; 95% CI: 0.955–1.956; p=0.088; **Table 3**). In the analysis of PSM data, the risk of death in those with sarcopenic obesity/overweight was also significantly higher (HR: 1.481; 95% CI: 1.203–1.823; p<0.001; **Table 3**).

**Table 3:** The hazard ratios for death in sarcopenic obesity/overweight compared to obesity/overweight with sufficient ASMI.

	Model 1		Model 2	
	HR (95% CI)	P-value	HR (95% CI)	P-value
Total	1.612 (1.328–1.957)	<0.001	1.481 (1.203–1.823)	<0.001
Subgroup by age				
Aged ≤ 65 years	2.707 (1.813–4.043)	<0.001		
Aged > 65 years	1.174 (0.982–1.404)	0.079		
Subgroup by sex				
Men	1.429 (1.101–1.854)	0.007		
Women	1.924 (1.435–2.581)	<0.001		
Subgroup by cause of death				
Cancer	1.541 (1.028–2.309)	0.036		
Non-cancer	1.629 (1.306–2.031)	<0.001		
CVD/CVA	1.367 (0.955–1.956)	0.088		

CVD, cardiovascular disease; CVA, cerebrovascular accident.

Model 1: Adjusted for age, sex, ethnicity/race, smoking status, alcohol consumption, history of cancer at baseline, diabetes mellitus, hypertension, dyslipidemia, and previous CVD events.

Model 2: Adjusted for diabetes mellitus, hypertension, dyslipidemia, and previous CVD events in propensity score-matched data for age, sex, ethnicity/race, smoking status, and alcohol consumption.

### The sensitivity analysis of the Cox proportional hazards model by time of death

**Tables 4** and **5** show the risk of all-cause mortality according to body composition indices and sarcopenic obesity/overweight status, respectively, considering the time of death. As shown in **Table 4**, ASMI and FMI were positively and negatively associated with the risk of all-cause mortality, respectively, independent of the time of death. However, when participants who died within the first 1, 2, and 3 years were excluded, the risk of all-cause mortality for sarcopenic obesity/overweight did not show a significant increase (**Table 5**).

**Table 4:** Sensitivity analysis of the Cox proportional hazards model for all-cause mortality by time of death and body composition indices in participants with obesity/overweight.

	ASMI		FMI	
	HR (95% CI)	P-value	HR (95% CI)	P-value
Primary analysis	0.856 (0.802–0.915)	<0.001	1.045 (1.021–1.069)	<0.001
Excluding patients with event within the first year	0.857 (0.802–0.917)	<0.001	1.040 (1.016–1.065)	0.001
Excluding patients with event within 2 years	0.861 (0.803–0.922)	<0.001	1.040 (1.015–1.065)	0.001
Excluding patients with event within 3 years	0.864 (0.804–0.928)	<0.001	1.040 (1.015–1.066)	0.002

ASMI, appendicular skeletal mass index; FMI, fat mass index; CVD, cardiovascular disease; CVA, cerebrovascular accident.

Adjusted for age, sex, ethnicity/race, smoking status, alcohol consumption, history of cancer at baseline, diabetes mellitus, hypertension, dyslipidemia, previous CVD events, and ASMI or FMI.

**Table 5:** Sensitivity analysis of the Cox proportional hazards model for all-cause mortality by time to death and sarcopenia status in obese/overweight participants.

	Sarcopenia	
	HR (95% CI)	P-value
Primary analysis	1.612 (1.328–1.957)	<0.001
Excluding patients with events within the first year	1.348 (0.895–2.030)	0.152
Excluding patients with events within 2 years	1.321 (0.864–2.020)	0.199
Excluding patients with events within 3 years	1.243 (0.791–1.954)	0.345

Adjusted for age, sex, ethnicity/race, smoking status, alcohol consumption, history of cancer at baseline, diabetes mellitus, hypertension, dyslipidemia, previous CVD events, and ASMI or FMI.

## Discussion

When food is consumed, it is converted into energy and used by the body; excess energy is converted into glycogen and triglycerides, which are stored in the body. Obesity develops when there is an imbalance between energy intake and expenditure. The risk factors for overweight/obesity include insufficient exercise, unhealthy eating habits, a lack of sufficient high-quality sleep, stress, diseases, genetics, and medications. Obesity is associated with diseases such as diabetes mellitus, CVD, stroke, hypertension, pulmonary disease, cancer, and NAFLD (non-alcoholic fatty liver disease) (32). Obesity is also associated with increased mortality (33). However, CVD-associated mortality decreases in the presence of obesity, a phenomenon known as the obesity paradox (34).

Sarcopenia is characterized by decreased muscle function and muscle mass (35). Muscle mass decreases by 3–8% every ten years after the age of 30 years, and this rate increases after 60 years of age (36). Sarcopenia has a prevalence of 6–15% in people aged > 65 years (37). The main causes of sarcopenia include reduced nutritional intake and activity, comorbidities, and drug use. Sarcopenia must be distinguished from malnutrition, cachexia, and senility before treatment. It is diagnosed based on reduced muscle strength, quantity, quality, and physical performance (30). Sarcopenia is associated with increased hospitalization duration, number of hospitalizations, and mortality (38).

This large-scale study aimed to determine the association between ASMI and FMI on mortality in participants with obesity. The HR for mortality increased as ASMI increased, which is consistent with previous studies suggesting that sarcopenia is associated with increased mortality (39, 40). In addition, the mortality risk increased proportionally with FMI, contrary to previous studies that found that being overweight was associated with decreased mortality (33, 41). However, this result is consistent with those of previous studies investigating the association between FMI, but not BMI, and mortality (42, 43). Furthermore, sarcopenia increases mortality risk in participants with obesity, reaffirming the importance of sarcopenia in this population (44). Therefore, it is reasonable to assume that high muscle mass and low fat mass rather than high BMI are associated with reduced mortality.

Since the term “sarcopenic obesity” was first used by Heber et al. in 1996, it has become increasingly important. The incidence of sarcopenic obesity has increased owing to an increase in the older population and the presence of obesity, and sarcopenia exacerbates the adverse effects of obesity (45). Sarcopenic obesity has several causes. Aging causes changes in the body’s composition. Fat mass increases with age, whereas muscle mass decreases after 40 years of age. The basal metabolic rate of older people is low because both the number and oxidative function of mitochondria in muscles are reduced. In addition, although the amount of thermogenic adipose tissue required for adaptive thermogenesis decreases (46), there is no significant change in the desire for food intake, which causes weight and fat gain. Hormones play a crucial role in muscle growth and strength. Testosterone, growth hormone, insulin, and thyroid hormones are associated with sarcopenia (47). Testosterone is a potent anabolic hormone that stimulates protein synthesis and inhibits protein degradation. Testosterone also increases the size of muscle fibers while inhibiting the differentiation of adipocyte progenitor cells. It also increases the levels of another anabolic hormone, insulin-like growth factor 1 (IGF-1), in the muscles. The growth hormone enhances protein turnover and muscle mass but does not increase muscle strength. Skeletal muscle is an important organ for insulin-induced glycemic control; thus, decreased muscle mass and quality lead to increased fat mass (48). Thyroid hormones are also involved in muscle growth and contraction (49). In older adults, the secretion of testosterone and growth hormones and the biological activity of thyroid hormones decrease, whereas insulin resistance increases, making them vulnerable to sarcopenic obesity (50). Adipose tissue inflammation is associated with sarcopenic obesity. Fat accumulation in the adipose tissue increases the release of free fatty acids and the production of leptin/monocyte chemoattractant protein 1 (MCP-1) and decreases adiponectin secretion. These adipokines recruit macrophages that undergo M1 polarization. Macrophages secrete tumor necrosis alpha (TNF-α), interleukin 6 (IL-6), and leptin, which are associated with the secretion of proinflammatory cytokines and decreased levels of IGF-1. Furthermore, TNF-α impedes mitochondrial biosynthesis and muscle development (51).

This study investigated the effect of obesity on mortality in a large sample and found that muscle and fat mass, but not BMI, were important for predicting mortality risk. ASMI was associated with a lower risk of mortality, independent of age and sex, while FMI was associated with a higher risk of mortality, independent of age, in men. Unexpectedly, in the PSM data, FMI was associated with a lower risk of mortality, which may reflect poor nutritional status in older patients with sarcopenia; however, further research is needed. In addition, when comparing participants with obesity and sarcopenia to participants with sarcopenia alone, the mortality rate was higher in the participants with obesity and sarcopenia. This effect was more significant in participants aged < 65 years. While other studies have investigated the effect of sarcopenia on mortality in older people (39, 40), this study revealed that sarcopenia was associated with increased mortality even in participants aged < 65 years. In the sensitivity analysis by time of death, when premature death was excluded, sarcopenic obesity/overweight was not associated with a significant increase in mortality, suggesting that sarcopenic obesity is associated with premature death. However, this study has several limitations. First, the effects of muscle strength or physical performance on mortality were not investigated. Secondly, the effects of sarcopenia management on mortality were not evaluated. Third, our study did not evaluate which type of fat (subcutaneous or visceral) influenced the mortality rate. Nevertheless, our study reaffirmed that sarcopenic obesity increases mortality rates.

## Conclusions

These findings highlight the importance of maintaining muscle mass and managing fat mass to reduce mortality risk among people who are obese or overweight. In particular, this study provides evidence that the obesity paradox is incorrect by revealing that sarcopenia increases the mortality risk in individuals who are obese or overweight. Further investigations are warranted for the management of sarcopenic obesity/overweight.

## Data availability statement

Publicly available datasets were analyzed in this study. This data can be found here: https://www.cdc.gov/nchs/nhanes/index.htm.

## Ethics statement

All NHANES protocols conducted within the United States were authorized by the Research Ethics Review Board of the National Center for Health Statistics, U.S. Centers for Disease Control and Prevention (NCHS IRB/ERB Protocol Number: 1999–2004, Protocol #98–12; 2005–2010, Protocol #2005–06; 2011–2016, Protocol #2011–17) under the declaration of Helsinki. All participants provided informed consent prior to the study.

## Author contributions

HL: Conceptualization, Formal analysis, Investigation, Methodology, Writing – original draft, Writing – review & editing. HC: Formal analysis, Investigation, Writing – review & editing. YK: Formal analysis, Investigation, Writing – review & editing. MC: Formal analysis, Investigation, Writing – review & editing. YR: Formal analysis, Investigation, Writing – review & editing. JY: Formal analysis, Investigation, Writing – review & editing. CO: Formal analysis, Investigation, Supervision, Writing – original draft, Writing – review & editing. JK: Formal analysis, Investigation, Supervision, Visualization, Writing – original draft, Writing – review & editing. SM: Conceptualization, Data curation, Formal analysis, Investigation, Methodology, Supervision, Visualization, Writing – original draft, Writing – review & editing.
